# Early Versus Late Drainage Removal in Patients Who Underwent Pancreaticoduodenectomy: A Comprehensive Systematic Review and Meta-analysis of Randomized Controlled Trials Using Trial Sequential Analysis

**DOI:** 10.1245/s10434-024-14959-w

**Published:** 2024-02-24

**Authors:** Claudio Ricci, Davide Giovanni Grego, Laura Alberici, Carlo Ingaldi, Stefano Togni, Ermenegilda De Dona, Riccardo Casadei

**Affiliations:** 1 Division of Pancreatic Surgery, IRCCS Azienda Ospedaliero-Universitaria Di Bologna, Bologna, Italy; 2 Department of Internal Medicine and Surgery (DIMEC); Alma Mater Studiorum, University of Bologna, Bologna, Italy

**Keywords:** Pancreatic surgery, Prophylactic drainages, Pancreatic fistula

## Abstract

**Background:**

The superiority of early drain removal (EDR) versus late (LDR) after pancreaticoduodenectomy (PD) has been demonstrated only in RCTs.

**Methods:**

A meta-analysis was conducted using a random-effects model and trial sequential analysis. The critical endpoints were morbidity, redrainage, relaparotomy, and postoperative pancreatic fistula (CR-POPF). Hemorrhage (PPH), delayed gastric emptying (DGE), length of stay (LOS), and readmission rates were also evaluated. Risk ratios (RRs) and mean differences (MDs) with a 95% confidence interval (CI) were calculated. Type I and type II errors were excluded, comparing the accrued sample size (ASS) with the required sample size (RIS). When RIS is superior to ASS, type I or II errors can be hypothesized.

**Results:**

ASS was 632 for all endpoints except DGE and PPH (557 patients). The major morbidity (RR 0.55; 95% CI 0.32–0.97) was lower in the EDR group. The CR-POPF rate was lower in the EDR than in the LDR group (RR 0.50), but this difference is not statistically significant (95% CI 0.24–1.03). The RIS to confirm or exclude these results can be reached by randomizing 5959 patients. The need for percutaneous drainage, relaparotomy, PPH, DGE, and readmission rates was similar. The related RISs were higher than ASS, and type II errors cannot be excluded. LOS was shorter in the EDR than the LDR group (MD − 2.25; 95% CI − 3.23 to − 1.28). The RIS was 567, and type I errors can be excluded.

**Conclusions:**

EDR, compared with LDR, is associated with lower major morbidity and shorter LOS.

**Supplementary Information:**

The online version contains supplementary material available at 10.1245/s10434-024-14959-w.

Complications after pancreaticoduodenectomy (PD) can be observed in more than 50% of patients^1^ and are frequently related to clinically relevant postoperative pancreatic fistula (CR-POPF).^2^ Abdominal drainage tube placement is an effective method for early recognition of CR-POPF and related complications, such as postpancrectomy hemorrhage (PPH).^3^ Moreover, abdominal drainage can help mitigate the negative consequences of POPF by early evacuation of pancreatic and enteric juice from the peritoneal cavity.^2^ In the enhanced recovery after surgery (ERAS) era, the role of prophylactic abdominal drainage in abdominal surgery has been questioned. Nowadays, the omission of drainage is encouraged in several major abdominal procedures, such as liver or colonic resection.^4,5^ However, the abandonment of prophylactic abdominal drainage seems unwelcome by pancreatic surgeons, who prefer early removal (EDR).^6^ The rationale of EDR is based on the hypothesis that retrograde bacterial infection through abdominal drainage tube could trigger CR-POPF.^7^ A recent meta-analysis^8^ supports the safety of the EDR approach, even though including eight nonrandomized clinical trials (RCTs) weakened the credibility of the results. A new meta-analysis, including only RCTs, was carried out to clarify this critical point. The trial sequential analysis (TSA) methodology was used to avoid false positive or false negative results owing to the small sample size.^9,10^ TSA analyses all published RCTs by including them chronologically, and estimate the required sample size (RIS) needed to accept or reject the null hypothesis without including type I or II errors.^11,12^

## Patients and Methods

The study protocol was preregistered in PROSPERO (CRD42023397030). Preferred Reporting Items for Systematic Reviews and Meta-Analyses checklist (PRISMA) was used to check the manuscript.^13^

### Eligibility Criteria

The Population Intervention Control Outcomes Study (PICOS) approach was used to define the inclusion and exclusion criteria.^14^ “Population” was represented by patients who underwent pancreaticoduodenectomy (PD). “Intervention” was considered the EDR. The removal was defined early when intrabdominal drainage(s) was removed by postoperative day 3 (POD3). “Control” group included any approach where the drainage removal started from postoperative day 5 (POD5). “Studies” were included only when the design was randomized.

### Information Source, Search, Study Selection, and Data Collection Process

The last search was carried out on 15 July 2023. The search string was managed using the systematic review (SR) accelerator^15^ and reported in the Supplementary Methods. The PubMed/Medline, Scopus, and Cochrane databases were used.

### Data Items

The following information was described for each study: authors, affiliation and country, year of publication, registration number, the type and the number of the drain(s), the characteristics of the pancreatic remnant, and the type of tumors. The importance of outcomes was evaluated using the Grading of Recommendations Assessment, Development, and Evaluation (GRADE) approach.^16^ Postoperative mortality, major morbidity defined according to the Clavien–Dindo system^17^ > grade II, need for percutaneous redrainage, CR-POPF according to the updated International Study Group of Pancreatic Fistula (ISGPF) definition^2^ and relaparotomy was considered “critical.” Postpancreatectomy hemorrhage (PPH),^3^ delayed gastric emptying (DGE),^18^ length of stay (LOS), and readmission were defined as “important” but not “critical.” Two authors (D.G.G. and E.D.D.) evaluated the quality of the included studies using the revised tool for assessing the risk of bias in randomized trials (RoB 2).^19^ Any disagreement was solved after a collegial discussion involving the first author (C.R.). All variables were described using frequencies and percentages or mean and standard deviations (S.D.). When the study reported median and interquartile ranges, a dedicated statistical algorithm was used to obtain mean and SD.^20,21^

### Summary Measures and Synthesis of Results

Two main measures were calculated: (i) the measure of risk association, such as the risk ratio (RR) and mean difference (MD), reported with a 95% confidence interval (CI) and (ii) the required information size (RIS). RIS is the a priori sample size that should be reached to obtain credible results without occurring in type I and type II errors.^6^ RIS is calculated by considering the heterogeneity of included studies and setting the threshold of type I errors at 5% and type II at 20%.^7^ The TSA data were reported in the Cartesian plane in which the *y*-axis indicates the *Z*-score while the *x*-axis is the cumulative sample size. *Z*-score is associated with the *P* value; when it is higher than 1.96, the *P* value is less than 0.05. The *Z*-curve is obtained by adding each study sequentially. The *Z*-curve can cross three thresholds: the conventional (dotted red horizontal lines), monitoring boundaries (dotted black logarithmic lines), and futility boundaries (dotted black lines). The conventional edge is to the nominal threshold of *P* value = 0.05. False-positive results (type I error) can be observed when the *Z*-curve crosses this limit, but RIS still needs to be obtained. The monitoring thresholds are the *Z*-scores at which type I errors are absent. On the contrary, a false-negative result (type II error) should be hypothesized when the *Z*-curve remains within conventional and monitoring threshold, and RIS is not yet reached.^8,9^ Finally, if the RIS is reached with no significant effect, type II error could be rejected. In this case, the *Z*-score is within the futility boundaries, representing the threshold for non-superiority and non-inferiority effects. In other words, any additional randomization is useless for finding differences between the two arms. The meta-analysis was carried out in line with recommendations from the Cochrane Collaboration,^22^ and the Mantel–Haenszel random-effects model was used to calculate effect sizes.^23^

### Risk of Bias Across Studies and Meta-regression Analysis

The heterogeneity was described using *I*^2^ and Cochran’s *Q* statistics.^24^ Heterogeneity was also calculated as diversity (*D*^2^).^25^ The effect of covariates was measured with a meta-regression analysis.^26,27^ The publication bias was evaluated using Egger tests,^28^ and a *P* value < 0.05 indicated a non-negligible “small-study effect.” Statistical analysis was carried out using dedicated packages for *R*. TSA was performed using the trial sequential analysis software.^9^

## Results

### Studies Selection, Characteristics, and Risk of Bias Within the Studies

The PRISMA flowchart is shown in Supplementary Fig. 1. The systematic search identified 18,501 potential articles: 6215 from the Medline/PubMed database, 6952 from the ISI Web of Science, and 5334 from the Cochrane database. After deduplication, 12,921 papers remained. Of these, 12,505 were excluded by screening the title and abstract because they were not pertinent to the study question. In total, 223 were reviewed in full-text form, and 220 were excluded. The paper by Bassi et al.^29^ was excluded for the impossibility of extracting the data about PDs. Finally, four studies were included in the analysis.^30–33^ The accrued sample size was 632: 317 (50.1%) in the EDR arm and 315 (49.8%) in the LDR arm. In Table 1, the characteristics of the included studies were reported.

**Table 1:** Characteristics of the four included studies

First author/year	Type of resection	Sample size	Affilation/country	Registration number	Drain characteristics			Soft remnant (RR)	Wirsung < 3 mm (RR)	PDAC (RR)	Rob2
					Closed system	Active suction	Number				
McMillan et al.^30^ 2015	PD	75	Multicenter (USA–Italy)	NR	No	No	Prefixed	NR	NR	NR	Some concerns
Dembiski et al.^31^ 2019	PD	141	Multicenter (France)	NCT01368094	Yes	Yes	Discretional	0.99	1.04	1.14	Low risk
Dai et al.^32^ 2020	PD	104	Peking union medical college hospital (China)	NCT02230436	No	No	Prefixed	0.50	0.95	0.87	Low risk
Dai et al.^33^ 2022	PD	312	Multicenter (China)	NCT03055676	Yes	No	Discretional	0.84	0.79	1.06	Low risk

*PD* pancreaticoduodenectomy, *DP* distal pancreatectomy, *RR* risk ratio (rate in “early” group/ rate in “late” group), *PDAC* pancreatic ductal adenocarcinoma, *CI* confidence interval

### Results of Individual Studies and Synthesis of the Results

**Fig. 1:**
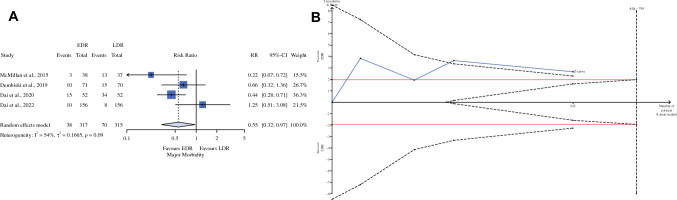
Major morbidity; **A**: forest plot; **B**: the *x*-axis is the number of patients yet to be randomized; the *y*-axis is the cumulative *Z*-score value representing the effect of each arm; the blue line is the cumulative *Z*-score obtained by combining the studies; and the dotted red horizontal lines are the conventional boundaries (*P* value < 0.05); when the *Z*-curve crosses the conventional boundaries, and the required information size (RIS) is not reached, the result is a false positive (type I error); when the *Z*-curve does not cross the conventional boundaries and RIS is not reached, the result is a false negative (type II error); the dotted black near-logarithmic lines are the monitoring boundaries; when the *Z*-curve crosses the monitoring boundaries, the result is a true positive; the inverse dotted black lines are the futility boundaries (area in which any further randomization is useful); *EDR* early drain removal, *LDR* late drain removal, *RR* risk ratio, *RIS* required information size

**Fig. 2:**
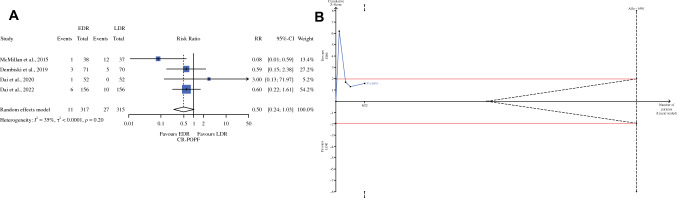
Clinically relevant POPF; **A**: forest plot; **B**: the *x*-axis is the number of patients yet to be randomized; the *y*-axis is the cumulative *Z*-score value representing the effect of each arm; the blue line is the cumulative *Z*-score obtained cumulating the studies; and the dotted red horizontal lines are the conventional boundaries (*P *value < 0.05); when the *Z*-curve crosses the conventional boundaries, and the required information size (RIS) is not reached, the result is a false positive (type I error); when the *Z*-curve does not cross the conventional boundaries and RIS is not reached, the result is a false negative (type II error); the dotted black near-logarithmic lines are the monitoring boundaries; when the *Z*-curve crosses the monitoring boundaries, the result is a true positive; the inverse dotted black lines are the futility boundaries (area in which any further randomization is useful); *EDR* early drain removal, *LDR* late drain removal, *RR* risk ratio, *RIS* required information size

Results regarding critical endpoints are presented in Table 2. The mortality rate was nil in four of five studies and, for this reason, was not analyzed. The major morbidity rate was lower (RR 0.55; 95% CI 0.32–0.97) in the EDR than in the LDR group (Fig. 1, panel A). The RIS of 798 still needs to be reached, but false-positive results can be excluded because the Z-line crosses the monitoring boundary (Fig. 1, panel B). The need for percutaneous drain placement was similar between the two arms (RR 0.75; 95% CI 0.24–2.35), and 23,787 additional patients should be randomized before excluding a false equivalence. Additionally, the rate of relaparotomy was similar between the two groups (RR 0.96, 95% CI 0.35–2.60). At the current RR, the RIS required to reject the null hypothesis without type II error was 42,952, indicating that 42,320 additional patients should be randomized to obtain credible information. POPF has a lower prevalence in EDR than LDR (Fig. 2, panel A), without statistical significance (RR 0.50; 95% CI 0.24–1.03). As shown in Fig. 2, panel B, the *Z*-curve showed that by randomizing 5959 patients, the “true” positive effect of EDR in reducing POPF could be demonstrated.

**Table 2:** Meta-analysis of critical and non-critical endpoints

Outcomes of interest	Event rate (%) or mean (SD)		RR or MD (95% CI)	*P* value	RIS	Δ	*C*-*Q*, *I*^2^ (%), *D* (%)	*P* value for reporting bias ^
	EDR	LDR						Egger
*Critical endpoints*								
Major morbidity	35/317 (11.9)	70/315 (22.2)	0.55 (0.32–0.97)	0.004	798	−166	0.009, 54, 42	0.858
percoutaneous drain	14/317 (4.4)	15/315 (4.8)	0.75 (0.24–2.35)	0.620	24,419	−23,787	0.210, 33, 33	0.675
Re-laparotomy	7/317 (2.2)	8/315 (2.5)	0.96 (0.35–2.60)	0.932	42,952	−42,320	0.755, 0, 21	0.725
POPF	11/317 (3.5)	27/315 (8.6)	0.50 (0.24–1.03)	0.060	6591	−5959	0.202, 35, 34	0.959
*Non-critical endpoints*								
PPH	8/279 (2.9)	9/278 (3.2)	0.96 (0.37–2.47)	0.934	80,263	−79,706	0.553, 0, 16	0.269
DGE	30/279 (10.8)	37/278 (13.3)	0.76 (0.35–1.64)	0.458	37,208	−36,651	0.458, 35, 31	0.786
Readmission	9/317 (2.8)	12/315 (3.8)	0.86 (0.24–3.01)	0.807	26,304	−25,672	0.176, 42, 32	0.493
LOS (days)	14.8 ± 6.4	16.9 ± 7	−2.25 (−3.23 to –1.28)	< 0.001	567	+65	0.534, 0, 17	0.918

Alternative hypothesis (H1): the placement and omission of drains have different results

Power: These data are the probability of rejecting a false null hypothesis (H0)

The pre-specified target value is 0.80

Alpha: the probability of rejecting a true null hypothesis

The pre-specified target value is 0.05

For the dichotomic endpoint, we use a different level of relative risk reduction (RRR), starting from the meta-analytic value; for the continuous value, we use an extra mean difference (MD) level, starting from the meta-analytic value

Null hypothesis (H0): the omission of drain guarantees similar results to drain placement

*SD* standard deviation, *RR* risk ratio, *MD* mean difference, *C-Q P* value of Cochran’s test, *I*^*2*^ higgins test, *D*^*2*^ diversity, *^* a reporting bias non-negligible is considered for *P* values < 0.10, *POPF* clinical relevant postoperative pancreatic fistula, *PPH* postpancreatectomy hemorrhage according to ISGPS classification, *DGE* delayed gastric emptying according to ISGPS classification, *LOS* length of stay, *–* not applicable

**Fig. 3:**
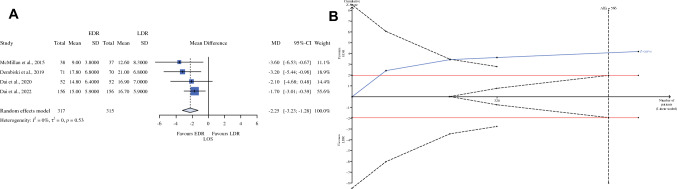
Length of stay; **A**: forest plot; **B**: the *x*-axis is the number of patients yet to be randomized; the *y*-axis is the cumulative *Z*-score value representing the effect of each arm; the blue line is the cumulative *Z*-score obtained combining the studies; and the dotted red horizontal lines are the conventional boundaries (*P* value < 0.05); when the *Z*-curve crosses the conventional boundaries, and the required information size (RIS) is not reached, the result is a false positive (type I error); when the *Z*-curve does not cross the conventional boundaries and RIS is not reached, the result is a false negative (type II error); the dotted black near-logarithmic lines are the monitoring boundaries; the result is a true positive when the *Z*-curve crosses the monitoring boundaries. The inverse dotted black lines are the futility boundaries (area in which any further randomization is helpful); *EDR* early drain removal, *LDR* late drain removal, *MD* mean difference, *RIS* required information size

PPH, DGE, and readmission are similar in the two groups, but a “false” equivalence cannot be excluded. LOS (Fig. 3, panel A) was significantly lower (MD − 2.25; 95% CI − 3.23 to − 1.28). The sample size is adequate, and the RIS was 567 (Fig. 3, panel B).

### Heterogeneity, Meta-regression Analysis, and Publication Bias

Significant heterogeneity was observed for major morbidity (*I*^2^ = 54%; *D* = 42%). Table 3 presented heterogeneity of major morbidity as not being influenced by PDAC, the texture of the pancreas, the type and number of drains, or the quality of the study.

**Table 3:** Meta-regression analysis

Confounding covariates	Effect on meta-analytical measures
	Major morbidity (RR) (95% CI)
Type of disease (RR PDAC)^	−5.65 (−23.63 to 12.34)
Type of pancreatic remnant (RR soft) ^	−2.12 (−10.23 to 5.98)
Type of drain (open vs closed)	0.65 (−1.64 to 2.94)
Active suction (no vs yes)	−1.17 (−3.66 to 1.32)
Number of drain (prefixed vs discretional)	−0.65 (−2.94 to 1.64)
Preregistered protocol (no vs yes)	1.51 (−1.59 to 4.62)

*RR* risk ratio, *MD* mean difference, *POPF* clinical relevant postoperative pancreatic fistula, *DP* distal pancreatectomy, *PD* pancreaticoduodenectomy, *^* the measure indicates that the change in meta-analytical measure was related to the increasing presence of covariate in “early” removal arm

## Discussion

The present study demonstrated that EDR after PD could have some advantages, reducing major morbidity and LOS in patients with low or intermediate risk of CR-POPF. These results were obtained by including only RCTs and using the TSA approach to exclude type I errors owing to inadequate sample size.

Regarding critical endpoints, TSA analysis showed that EDR, compared with LDR, halves the rate of major complications. This effect seems well demonstrated without needing further RCTs because an adequate sample size has yet to be reached, as shown in the TSA graph. In addition, CR-POPF seems reduced in the EDR group but without conventional statistical significance. In other words, the certainty of this effect is weak owing to imprecision because the 95% CI crosses the null effect line. However, observing the TSA plot, it becomes evident that the *Z*-score curve could simultaneously cross the conventional and monitoring boundaries by adding only a few hundred patients. Thus, in the following years, with one or two new RCTs, the definitive demonstration that EDR reduces the POPF could be obtained. There are several potential explanations to support these results. First, major morbidity in pancreatic surgery mainly depends on POPF and POPF-related events. Thus, EDR could have a similar effect on major morbidity and POPF. LDR removal could facilitate the development of POPF-related complications, because drainage could mechanically contribute to soft tissue and vessel corrosion owing to pancreatic enzymes.^34^ Moreover, retrograde and ectopic bacteria could invade the fistula area by extending the catheter time, triggering hemorrhage or anastomosis disruption.^35^ Several studies have demonstrated that delaying drainage removal causes an increased rate of bacterial-positive cultures.^7,36^ Retrograde infection could be the basis of CR-POPF infection in some POPFs after PD, mainly when the amylase originated from the branch duct rather than when originating from a disrupted Wirsung-jejunal anastomosis. However, the impact of EDR on POPF is small and requires several patients to be demonstrated.

Concerning relaparotomy and the need for percutaneous redrainage placement, the two approaches appear similar, even if the rarity of these events (< 2% and 5 %) makes it impossible to demonstrate the absence of type II errors. Several thousand patients should be randomized before excluding a false equivalence between the two approaches. Furthermore, the large RISs suggested that the differences, if present, are too small to be clinically irrelevant. The present study confirmed the safety of the EDR approach regarding important endpoints, suggesting that PPH, readmission, and DGE rates were similar. PPH and readmission are rare events, and the statistical demonstration of the equivalence or non-equivalence could be time consuming, requiring several patients and useless from the clinical point of view. DGE, from a physiopathological point of view, is historically attributed to the presence of clinically relevant POPF rate. Thus, assuming this rationale, it seems logical to expect a parallel reduction of DGE in the EDR arm. However, the results suggested that DGE risk was similar among the groups. This counterintuitive result can be explained by recent evidence demonstrating that not all DGEs are related to POPF presence.^37^ Finally, the reduction of LOS in the EDR arm is statistically significant, not a risk of type I error, and clinically relevant (nearly 2 days less than LDR patients). This result seems consistent with the reduction of major morbidity.

Our study has both strengths and limitations. First, the present meta-analysis is the first to include only RCTs. All included studies are homogenous as inclusion criteria, clearly defining the target population: patients with low-intermediate risk of POPF without clinical, laboratory (amylase values > 5000 U/mL), or radiological suspicion of clinically relevant POPF with POD3. Second, the meta-analytical results were validated using the TSA approach that permits obtaining a measure of the imprecision, namely classical *P* value, and the credibility of the results in estimating the presence of type I or II errors was validated. Moreover, TSA could help pancreatic surgeons in evaluating whether it is logical and helpful to plan further randomized trials, calculating the correct sample size, and the proper endpoints. Finally, the study included only PD, reducing the bias owing to including distal pancreatectomies.

Nevertheless, some limitations should be recognized. Firstly, a significant heterogeneity weakened major morbidity and POPF results. Even if metaregression explained some sources of RR variability, several covariates, such as the size of Wirsung, surgeons’ experience, or hospital volume, cannot be directly analyzed. Even if the CR-POPF affected both procedures, the severity of complications and the feasibility of rescue strategies, such as percutaneous drainage, would differ between the two types of resection. Another limitation was that the study included studies from different countries with different healthcare systems. Finally, some limitations are due to the TSA methodology, which remains a retrospective method to analyze the RCTs, and thus has the risk of data-driven hypotheses. Moreover, the TSA is a complex statistical approach that is challenging for clinicians.^38^

In conclusion, EDR, compared with LDR, is associated with lower major morbidity and shorter LOS. These results are robust and not at risk of type I errors. The target population in which EDR can be useful and safe is with patients who, by POD3, did not present clinical, biochemical, or radiological suspicions of clinically relevant POPF. An effect of EDR in reducing POPF could be present, but some high-quality, well-designed RCTs are needed to confirm these results. The number of patients that should be randomized to obtain a definitive conclusion is reasonable and achievable in a relatively short time.

### Supplementary Information

Below is the link to the electronic supplementary material.

Supplementary file1 (DOCX 138 kb)
