# Supplementary material for: Early Versus Late Drainage Removal in Patients Who Underwent Pancreaticoduodenectomy: A Comprehensive Systematic Review and Meta-analysis of Randomized Controlled Trials Using Trial Sequential Analysis
Source: Ann Surg Oncol. 2024 Feb 24;31(5):2943–50. doi: 10.1245/s10434-024-14959-w (PMC10997728; doi:10.1245/s10434-024-14959-w)
Supplement: Supplementary file 1 — Supplementary file1 (DOCX 138 kb) [file 10434_2024_14959_MOESM1_ESM.docx]

**Supplementary Methods**

The search string was the following: (‘Pancreaticoduodenectomy’[MeSH] OR ‘Pancreatectomy’[MeSH] OR ‘pancreatic diseases/surgery’ [MeSH] OR pancreatectomy [tiab] OR pancreato-duodenectomy [tiab] OR pancreaticoduodenectomy [tiab] OR pancreatic resection [tiab] OR pancreas resection [tiab]) AND (‘Drainage’[MeSH] OR drain [tiab] OR drains [tiab] OR drain* [tiab] OR suction [tiab]) AND ((randomized controlled trial [pt] OR controlled clinical trial [pt] OR randomized [tiab] OR clinical trials as topic [mesh: noexp] OR randomly [tiab] OR random [tiab] OR random* [tiab] OR trial [ti]) NOT (animals [mh] NOT humans [mh])). The string was built by combining keywords and MeSH and using the following three concepts: abdominal drainage, pancreatic surgery, and postoperative outcomes.

**Supplementary Figure 1 – PRISMA flowchart**

**
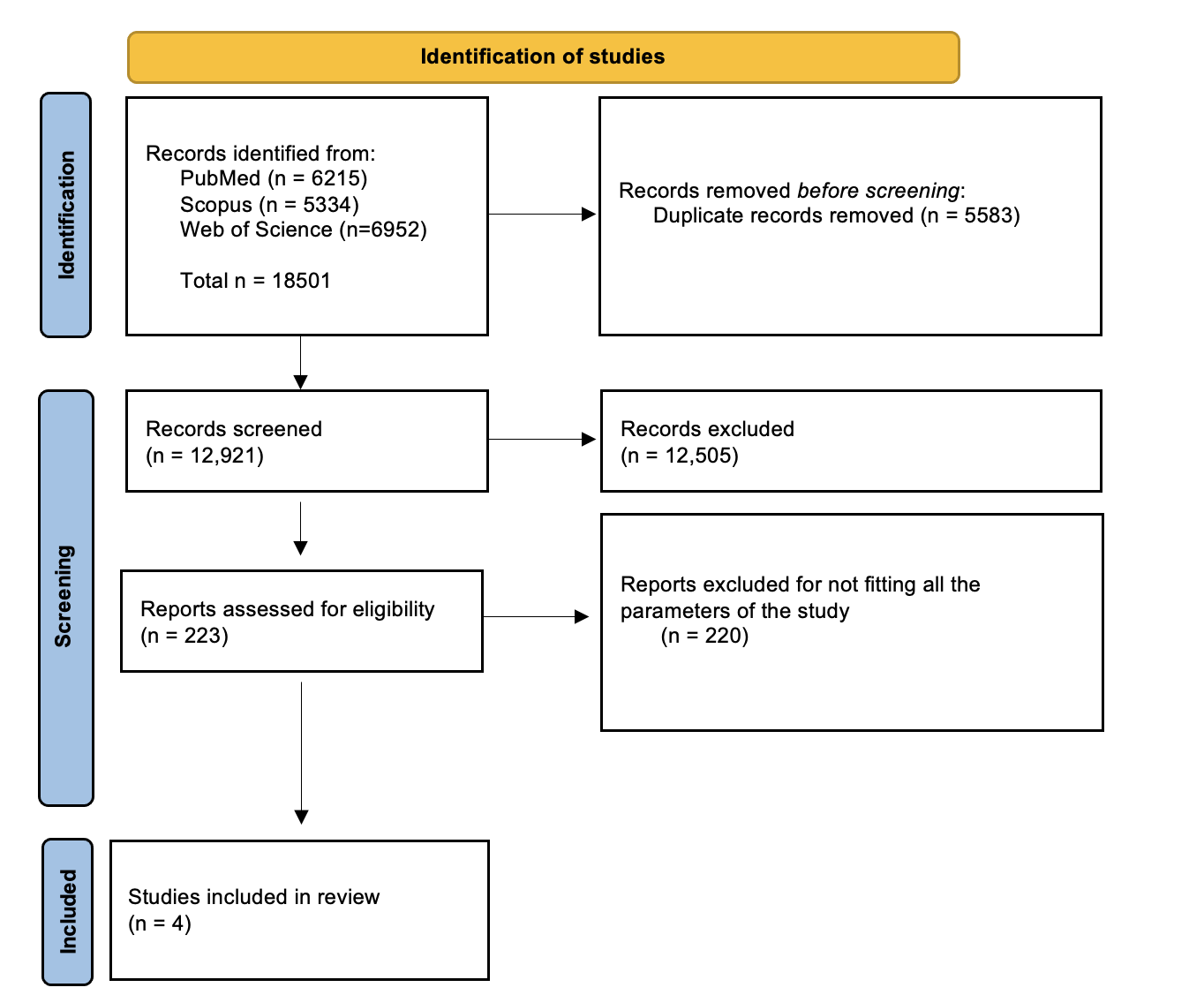
**
